# Supplementary material for: Modeling ETBF-Mediated Colorectal Tumorigenesis Using AOM/DSS in Wild-Type Mice
Source: Int J Mol Sci. 2025 Jun 27;26(13):6218. doi: 10.3390/ijms26136218 (PMC12249562; doi:10.3390/ijms26136218)
Supplement: Supplementary file 1 [file ijms-26-06218-s001.zip › ijms-3663744-supplementary.pdf]

[Supplementary data]

Modeling ETBF-Mediated Colorectal Tumorigenesis Using AOM/DSS in Wild-Type Mice

Figure S1. Comparable ETBF colonization in AOM/DSS-treated mice.

Figure S2. DSS dosage does not affect ETBF colonization in AOM/DSS-treated mice.

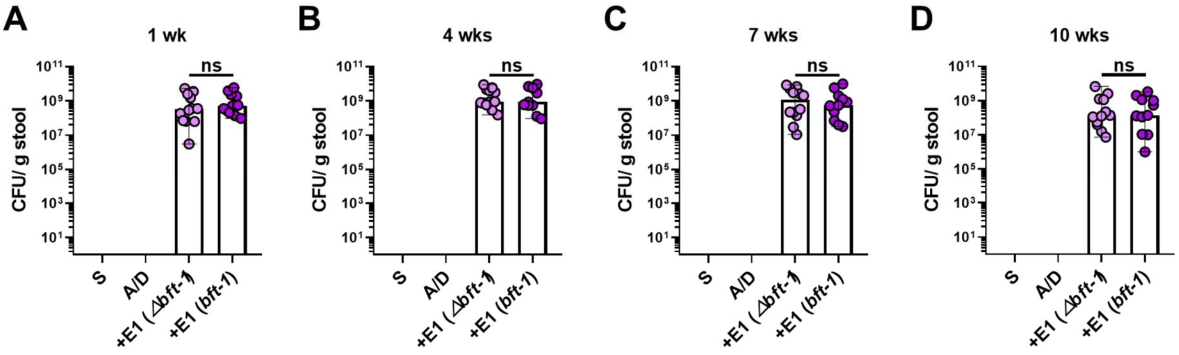

Figure S1. Comparable ETBF colonization in AOM/DSS-treated mice.

Stool samples from BALB/c female mice inoculated with ETBF (~1 × 10^9 CFU) were plated to determine the level of ETBF colonization. Colonization, typically between 10^7 and 10^9 CFU/g stool, was measured at 1 (A), 4 (B), 7 (C), and 10 (D) weeks post-infection. Results are presented as the mean ± SEM from three independent experiments. Experimental groups included sham controls (S), AOM/DSS-treated mice (A/D), and AOM/DSS-treated mice receiving WT-ETBF (bft-1) (+E1). Each data point represents a single mouse. Significance between treated groups was determined using Mann–Whitney *U* test. ns, no statistical significance.

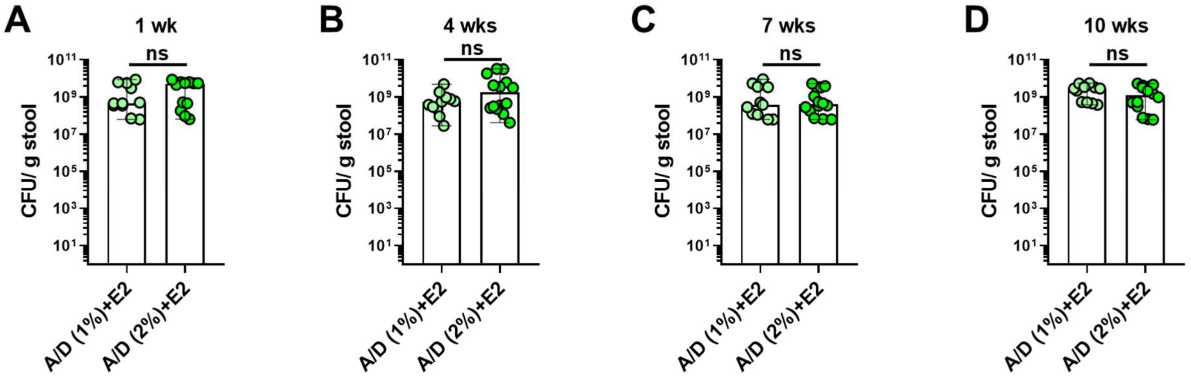

Figure S2. DSS dosage does not alter ETBF colonization in mice.

Stool samples from BALB/c female mice inoculated with ETBF (~1 × 10^9 CFU) were plated to determine the level of ETBF colonization. Colonization, typically between 10^7 and 10^9 CFU/g stool, was measured at 1 (A), 4 (B), 7 (C), and 10 (D) weeks post-infection. Results are presented as the mean ± SEM from three independent experiments. Experimental groups included sham controls (S), AOM/DSS-treated mice (A/D), and AOM/DSS-treated mice receiving WT-ETBF (*bft-2*) (+E2). Each data point represents a single mouse. Significance between treated groups was determined using Mann–Whitney *U* test. ns, no statistical significance.
